# Cost-effectiveness analysis of hepatocellular carcinoma screening by combinations of ultrasound and alpha-fetoprotein among Alaska Native people, 1983–2012

**DOI:** 10.3402/ijch.v75.31115

**Published:** 2016-05-18

**Authors:** Prabhu P. Gounder, Lisa R. Bulkow, Martin I. Meltzer, Michael G. Bruce, Thomas W. Hennessy, Mary Snowball, Philip R. Spradling, Bishwa B. Adhikari, Brian J. McMahon

**Affiliations:** 1 Arctic Investigations Program, Division of Preparedness and Emerging Infections, National Center for Emerging and Zoonotic Infectious Disease, U.S. Centers for Disease Control and Prevention (CDC), Anchorage, AK, USA; 2 Health Economics and Modeling Unit, Division of Preparedness and Emerging Infections, National Center for Emerging and Zoonotic Infectious Disease, U.S. Centers for Disease Control and Prevention, Atlanta, GA, USA; 3 Liver Disease and Hepatitis Program, Alaska Native Tribal Health Consortium, Anchorage, AK, USA; 4 Division of Viral Hepatitis, National Center for HIV/AIDS, Viral Hepatitis, STD, and TB Prevention Division of HIV/AIDS Prevention Centers for Disease Control and Prevention, Atlanta, GA, USA

**Keywords:** Alaska Native people, clinical outcome, diagnosis, early detection of cancer, economics

## Abstract

**Background:**

The American Association for the Study of Liver Diseases (AASLD) recommends semi-annual hepatocellular carcinoma (HCC) screening using ultrasound (US) in persons with chronic hepatitis B (CHB) virus infection at high risk for HCC such as Asian males aged ≥40 years and Asian females aged ≥50 years.

**Objective:**

To analyse the cost-effectiveness of 2 HCC screening methods in the Alaska Native (AN) health system: US-alone, or screening by alpha-fetoprotein (AFP) initially and switching to US for subsequent screenings if AFP >10 ng/mL (AFP→US).

**Design:**

A spreadsheet-based model was developed for accounting the costs of 2 hypothetical HCC screening methods. We used epidemiologic data from a cohort of 839 AN persons with CHB who were offered HCC screening by AFP/US semi-annually during 1983–2012. We assumed that compared with AFP→US, US-alone identifies 33% more tumours at an early stage (defined as a single tumour ≤5 cm or ≤3 tumours ≤3 cm in diameter). Years of life gained (YLG) attributed to screening was estimated by comparing additional years of survival among persons with early- compared with late-stage tumours. Screening costs were calculated using Medicare reimbursement rates in 2012. Future screening costs and YLG were projected over a 30-year time horizon using a 3% discount rate.

**Results:**

The total cost of screening for the cohort by AFP→US would have been approximately $357,000 ($36,000/early-stage tumour detected) compared to $814,000 ($59,000/early-stage tumour detected) by US-alone. The AFP→US method would have yielded an additional 27.8 YLG ($13,000/YLG) compared with 38.9 YLG ($21,000/YLG) for US-alone. Screening by US-alone would incur an additional $114,000 per extra early-tumour detected compared with AFP→US and $41,000 per extra YLG.

**Conclusions:**

Although US-alone HCC screening might have yielded more YLG than AFP→US, the reduced costs of the AFP→US method could expand access to HCC screening in resource constrained settings.

The World Health Organization estimates that 360 million persons live with chronic hepatitis B virus infection (CHB) worldwide (1). Persons with CHB are at increased risk for developing hepatocellular carcinoma (HCC) (2). The American Association for the Study of Liver Diseases (AASLD) guidelines recommend HCC screening when the risk exceeds 0.2% year within a population group, such as among Asian males aged >40 years and Asian females aged >50 years (3). The optimal method for HCC screening remains debatable (4). AASLD guidelines for managing CHB recommend screening of persons at high risk for HCC by ultrasound (US) every 6–12 months, but list serum alpha-fetoprotein (AFP) as an acceptable alternative in areas of the United States where US is not readily available (5). Conversely, separate AASLD guidelines for managing HCC state that AFP lacks sufficient sensitivity/specificity to serve as a useful screening test and, therefore, recommend screening every 6 months by US alone (3).

Alaska Native (AN) persons with CHB have an annual risk of 0.26% for developing HCC and might benefit from semi-annual HCC screening (3,6). Alaska is a large sparsely populated state, and a substantial proportion of AN people live in small villages that are inaccessible by road. In villages without an US facility, the costs associated with transporting persons by air to an US-capable regional health centre are an important barrier to HCC screening by US alone. For that reason, the Alaska Native Tribal Health System has screened all persons with CHB for HCC by semi-annual serum AFP measurements; only persons with an elevated AFP and persons with a family history of HCC or cirrhosis were referred for US.

Between 1982 and 1987, more than 53,000 AN persons were screened for CHB in a mass vaccination campaign (7); 1,535 persons tested positive for hepatitis B surface antigen during and after the campaign. All persons testing positive for hepatitis B surface antigen were offered semi-annual screening for HCC through the Alaska Native Tribal Health System. In total, 1,375 AN persons with CHB provided consent to enroll in a prospective cohort study that followed participants from January 1983 to December 2012. This prospective study was approved by the Institutional Review Boards of the Alaska Native Tribal Health Consortium and the Centres for Disease Control and Prevention. For this present study, we used data from the prospectively studied cohort of AN persons with CHB to compare the cost-effectiveness of HCC screening by 2 methods: screening by US semi-annually (US-alone method) versus semi-annual screening by AFP alone and switching to US alone if an AFP >10 ng/m was detected (AFP→US method). Our analysis can guide public health officials on choosing the optimal HCC screening method for persons with CHB living in resource constrained settings where US is not available, and the cost of transportation to an US-equipped facility can be a barrier.

## Methods

We built a spreadsheet-based model to compare the cost-effectiveness of HCC screening by US-alone and AFP→US methods. For the US-alone method, we assumed patients would have received an US at every screening opportunity. For the AFP→US method, we assumed that all persons would have been screened by AFP initially and switched to US for all subsequent screening opportunities if AFP >10 ng/mL on at least 1 measurement. Based on AASLD guidelines criteria (3), we assumed that HCC screening in the prospectively followed cohort of AN persons with CHB began for all males and females once they attained ages ≥40 and ≥50 years, respectively. Under both screening methods, we assumed that HCC screening continued until patient death or the end of the study period (December 31, 2012).

### Epidemiologic data

For this cost-effectiveness analysis, we used data from AN patients with CHB who participated in the prospective cohort study during 1983–2012 (see Appendix Table I for cohort participants’ demographic and clinical characteristics). We reviewed the medical records for cohort participants to determine the: (a) dates and results of all AFP measurements, (b) date of HCC diagnosis, and (c) date of death. For persons who developed HCC, the dates and results of all liver imaging studies were obtained from the medical records. Additionally, we cross-referenced the names of persons in the study cohort with the Alaska Native Tumour Registry, a participant of the National Cancer Institute Surveillance, Epidemiology, and End Results Program, to identify persons with HCC (8). Cohort participants who died or developed HCC <6 months after cohort entry were excluded from our analysis because they likely would not have benefited from either screening method. In total, 564 males attained age ≥40 years and 275 females attained age ≥50 years during 1983–2012 (the age/sex target group for HCC screening) and their data provided epidemiologic input for our model (Table I). Among cohort participants included in our analysis, 21 were diagnosed with HCC ≥6 months after cohort entry.

**Table I:** Epidemiologic data for a cost analysis from a prospectively followed cohort of AN persons with chronic hepatitis B virus infection, 1983–2012

Epidemiologic input	Data
Number of cohort participants^a^	839
Males (%)	564 (67%)
Number of cohort participants with HCC (%)	21 (2.5%)
Early-stage tumour^b^	10
Median (mean) years survival by tumour stage	
Early stage^b^	3.1 (5.0)
Late stage	0.2 (0.8)
Total number of potential screening opportunities^c^	21,226
Total number of AFP measurements in cohort^d^	10,931
Median AFP measurements/person	11
% of total potential screening opportunities	51%

AFP, alpha-fetoprotein; HCC, hepatocellular carcinoma; US, ultrasound.

a Comprises males attaining age ≥40 years and females attaining age ≥50 years during January 1, 1983 to December 31, 2012.

b Single tumour ≤5 cm in diameter or ≤3 tumours ≤3 cm in diameter.

c Assumes persons received screening for HCC every 6 months.

d Assumes persons received screening for HCC on dates for which an AFP measurement was documented (excluding AFP measurements occurring <4 months after a prior measurement).

### Tumour stage classification

The HCC tumour size was determined by either US or computed tomography (CT). If the number of tumours or tumour sizes were discrepant between ultrasound and CT for the same patient, we used the CT imaging results. We categorised patients with HCC as having an early-stage tumour as defined by the Milan criteria as follows: single tumour ≤5 cm in diameter or ≤3 tumours each ≤3 cm in diameter, and no vascular or lymph node invasion (9). Tumours not meeting Milan criteria were categorised as late-stage. To be conservative and not overestimate potential benefits of HCC screening, we also categorised as late-stage those tumours of unknown size (for patients who declined additional evaluation).

### Estimating the number of early-stage tumours identified by each screening method

Neither the AFP→US method nor the US-alone method was used in practice in the prospectively followed cohort. Therefore, we had to make assumptions to estimate the number of HCC tumours that would have been identified at an early stage by the 2 hypothetical screening methods (AFP→US and US-alone) based on the results of the actual HCC screening method used in the prospectively followed cohort during 1983–2012. For the actual HCC screening method, all AN patients with CHB were offered screening by AFP every 6 months regardless of age or risk factors for developing HCC and only those patients at high risk for HCC or with an elevated AFP were referred for US. Because the hypothetical AFP→US method closely resembles the actual screening method, we assumed the AFP→US method would have identified the same number of early-stage tumours as the actual screening method (Tables I and II).

**Table II:** Base-case model assumptions for estimating the costs for hepatocellular carcinoma screening

Model input	Assumption
Number of screening tests performed by AFP→US^a^	10,931
Number of AFP measurements	9,378
Number of US screenings	1,553
Number of ultrasounds performed by US-alone method^b^	10,931
Tumours detected at an early stage by AFP→US method^c^	10
Tumours detected at an early stage by US-alone method according to size^c^,^d^	14
Direct costs per test	
(Medicare reimbursement rates in 2012)	
AFP	$109.94
US	$26.76
Roundtrip cost/patient to an US-equipped facility^e^	$200
% cohort members requiring transportation to US facility	60%

AFP, alpha-fetoprotein; HCC, hepatocellular carcinoma; US, ultrasound.

a Assumes patients received screening for HCC by serum AFP measurements initially and switched to ultrasound for all subsequent screenings if AFP >10 ng/mL.

b Assumes patients received screening for HCC by ultrasound on dates for which AFP measurements are recorded.

c Single tumour ≤5 cm in diameter or ≤3 tumours ≤3 cm in diameter.

d Assumes 33% (4) of tumours identified at a late stage by the AFP→US method were identified by the US-alone method at an early stage.

e For patients living in rural Alaska areas without ready access to US.

In order to determine the number of early-stage tumours detected by the hypothetical US-alone method, we assumed that approximately 33% of the late-stage tumours identified by the actual screening method in the cohort would instead have been identified at an early stage by the US-alone method. This assumption was based on a previous study demonstrating that the sensitivity of AFP >10.9 ng/mL for detecting early-stage HCC was 66%, and on a meta-analysis which reported a pooled sensitivity for ultrasound of 63% for detecting an early-stage HCC and 94% for detecting any tumour before becoming clinically apparent (10,11). Although the sensitivity of US to detect HCC tumours can be lower in certain circumstances, such as in persons with cirrhosis, we opted to use the best-case scenario US sensitivity of 94% when comparing with AFP because guidelines presume that US is superior to AFP for HCC screening (3,5,12).

### Estimating survival by tumour stage

For both hypothetical screening approaches, we calculated the additional years of life gained (YLG) in the cohort of 839 persons with CHB compared with no screening. We assumed that the median (mean) years of survival among persons with late-stage HCC tumours represented survival with no screening. We further assumed that the additional YLG at median (mean) among persons with early-stage tumours were the result of screening (Table I). All persons within a tumour-stage category were assumed to have the same median (mean) survival by either the AFP→US approach or the US-alone approach (Table I); only the number of persons within each tumour-stage category differed between the 2 approaches (Table II). We calculated the additional median YLG attributable to screening as follows:

Additional YLG at median survival=[(# early-stage tumours)×(survival in early-stage tumour)] – [(# late-stage tumours)×(survival in late-stage tumours)].

### Total number of screening tests performed

We estimated the number of screening tests that would have been performed in the 839 cohort participants meeting age/sex criteria for HCC screening during the 30-year time horizon of our study by assuming that screening occurred only on dates of when documented AFP tests were actually performed. We further assumed that it would be unlikely that a person failing to have their blood drawn would instead comply with a recommendation to obtain an US. We thus assumed that the total number of documented AFP measurements also represents the potential US screening opportunities. We then determined the total number of AFP and US tests that would have been performed under the 2 hypothetical screening methods.

Assuming that all cohort members received a screening test for HCC every 6 months, there were a total of 21,226 screening opportunities in the cohort during the 30-year time horizon (Table I). However, AFP measurements were documented for only 10,931 (51.4%) screening opportunities (median: 11 HCC screening tests/person). By the AFP→US method, screening would have occurred by measuring serum AFP at 9,378 opportunities and by US at 1,553 opportunities among persons with at least 1 elevated serum AFP (Table II). By the US-alone method, we assume that an US would have been performed at all 10,931 screening opportunities (Table II).

### Cost analysis

We estimated direct costs of screening from the payer's perspective, the Alaska Native Health System. We used 2012 Medicare reimbursement rates in Alaska for AFP and US ($26.76 and $109.94 per test, respectively) to calculate the cost of screening at our institution (based on personal communication, Alaska Native Medical Center). For the 60% of patients living in communities without an US facility, we estimated that air transportation to the nearest regional health centre costs on average $200 per person per round trip. We also compared the 2 screening methods in terms of the cost/tumour detected by summing the cost of all AFP and US measurements that would have been performed in the cohort under each screening method and dividing by the number of tumours detected at an early stage in the cohort. We did not account for treatment costs after early- or late-tumour detections because we assumed that treatment costs will be similar irrespective of the methods used to detect tumour.

### Cost-effectiveness calculations

We evaluated the cost-effectiveness of the AFP→US and US-alone approaches in terms of the cost/tumour detected and the cost/YLG. The cost/tumour detected was calculated by summing the cost of all AFP and US measurements that would have been performed in the cohort under each screening approach and dividing by the number of early-stage tumours detected in the cohort. The cost/YLG was calculated by summing the cost of all AFP and US measurements that would have been performed in the cohort under each screening approach and dividing by the total number of YLG under each approach, respectively. The cost/YLG was calculated by using both the both the mean and median estimates for YLG. Finally, to account for differences in time between when screening costs may occur and health benefits are obtained, we discounted future costs and benefits (e.g. health outcomes such as YLG) to reference year 2012 at 3% year over a time horizon of 30 years (reflecting the time period our prospectively followed study cohort was followed) (13).

### Sensitivity analysis

We performed a sensitivity analysis for the cost/early-stage tumour detected under both hypothetical screening methods by varying the percentage of late-stage tumours identified by AFP→US that would have been identified by US-alone at an early stage from 0% (indicating that the 2 methods had the same sensitivity) to 100% (indicating that US-alone identified all tumours in the cohort at an early stage). The base-case analysis assumed approximately 33% difference between the 2 methods.

We also considered the impact if all persons in our cohort had received HCC screening every 6 months as recommended. Of the 21,226 potential screening opportunities in the cohort, only 10,931 screenings occurred (approximately 51% of potential screenings) (Table I).

## Results

To summarise key input data for our cost analysis model (Tables I and II), the median survival among persons with early-stage HCC tumours was 3.1 years (minimum [min]=0.3 years; maximum [max]=14.2 years), and for late-stage tumours was 0.2 years (min=0 years; max=2.3 years). Of the 21 tumours that occurred in the prospectively followed cohort, 10 (47%) might have been identified by AFP→US at an early stage. Assuming that approximately 33% of tumours identified at a late stage by AFP→US were instead detected by the US-alone method at an early stage, then 14 (67%) tumours might have been detected at an early stage by US-alone.

Our base-case analysis indicates that US-alone alone would have yielded more total YLG compared with AFP→US. Undiscounted YLG in the cohort based on median survival by US-alone was estimated at 41.4 years compared with 29.6 years by AFP→US (Table III). However, the undiscounted total direct cost of the screening program, without including transportation costs, during the study period would have been $1.2 million by US-alone compared to $528,000 by AFP→US. Discounting at 3%/year reduced those costs to $357,000 and $814,000, respectively. The approximate undiscounted cost/YLG without including transportation costs was lower for AFP→US ($18,000) than US-alone ($39,000) (discounted approximately $13,000 and $21,000, respectively). The total cost of the screening program in the cohort, the cost/tumour detected, and the cost/YLG at median/mean survival by AFP→US remained lower than US-alone after accounting for travel expenditure (Table III).

**Table III:** Comparing the costs of 2 hypothetical screening scenarios for hepatocellular carcinoma (HCC) – Alaska, 1983–2012^a^

	Costs/benefits without transportation expenses^b^		Costs/benefits with transportation expenses^b^	
		
	AFP→US^c^,^d^	US-alone^d^	AFP→US^c^,^d^	US-alone^d^
Analysis without discounting				
Total cost for cohort (Base Year 2012)	$528,000	$1,203,000	$868,000	$2,517,000
No. of early-tumours detected^e^	10	14	10	14
Median (mean) YLG for Cohort	29.6 (42)	41.4 (58.8)	29.6 (42)	41.4 (58.8)
Cost/early-stage tumour detected	$53,000	$86,000	$87,000	$180,000
Cost/YLG at median (mean)	$18,000 ($13,000)	$29,000 ($20,000)	$29,000 ($21,000)	$61,000 ($43,000)
Incremental cost-effectiveness ratios^f^				
Extra cost ($)/extra early-tumour detected	$169,000		$412,000	
Extra cost ($)/Extra YLG at Median (Mean)	$57,000 ($40,000)		$139,000 ($98,000)	
Analysis with discounting^g^				
Total cost for cohort (Base year 2012)	$357,000	$814,000	$587,000	$1,702,000
No. of early-tumours detected^e^	10	14	10	14
Median (mean) YLG for Cohort	27.8 (38.1)	38.9 (53.3)	27.8 (38.1)	38.9 (53.3)
Cost/early-stage tumour detection	$36,000	$58,000	$59,000	$122,000
Cost/YLG at median (mean)	$13,000 ($9,400)	$21,000 ($15,000)	$21,000 ($15,000)	$44,000 ($32,000)
Incremental cost-effectiveness ratios^f^				
Extra cost ($)/extra early-tumour detected	$114,000		$279,000	
Extra cost ($)/extra YLG at median (mean)	$41,000 ($30,000)		$100,000 ($73,000)	

AFP, alpha-fetoprotein; HCC, hepatocellular carcinoma; US, ultrasound; YLG, years of life gained.

a In a cohort of 839 hepatitis B virus infected AN men aged ≥40 years and women aged ≥50 years.

b Total costs rounded to the nearest thousand.

c Assumes patients received screening for HCC by serum AFP measurements initially and switched to ultrasound for all subsequent screenings if AFP >10 ng/mL.

d Assumes patients received screening for HCC on dates for which AFP measurements were recorded.

e Early-tumour if single tumour ≤5 cm in diameter or ≤3 tumours ≤3 cm in diameter; model assumes 33% (4) of tumours identified at a late stage by the AFP→US method were identified by the US-alone method at an early stage.

f Ratio=(costs US-alone – costs AFP→US)/(outcome US-alone – outcome AFP→US), where outcomes are the number of early-tumours detected or number YLG by early detection. Treatment costs after the detection of tumour (early or late) are not included in these estimates.

g Discounted direct costs of screening and YLG at 3%/year (reference year 2012) over a 30-year time horizon.

Compared to the AFP→US method, the US-only method had an incremental cost of $100,000 per each additional YLG and $279,000 per each additional early-stage tumour detected (discounted costs including transportation costs). Excluding transportation costs reduced this to $41,000 per additional YLG and $114,000 per each additional early-stage tumour detected (Table III).

### Sensitivity analysis

**Fig. 1:**
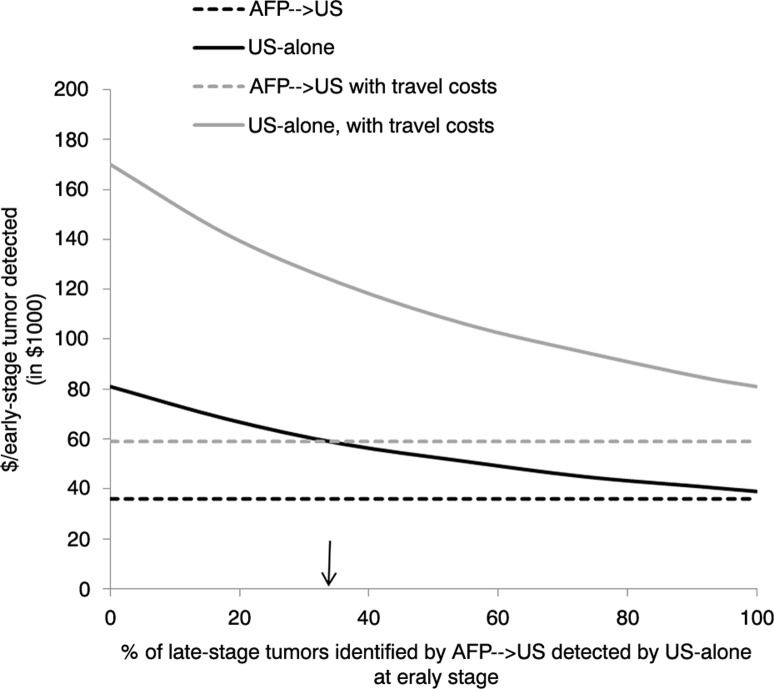
Sensitivity analysis: Impact of varying the percentage of hepatocellular carcinoma tumours that were identified by AFP→US at a late stage and potentially identified by US-alone at an early stage in hepatitis B virus infected AN persons.*^†‡§¶^ Abbreviations: AFP, alpha-fetoprotein; US, ultrasound; ↓, indicates base-case assumption *Screening assumed to start for men at age ≥40 years and for women at age ≥50 years. ^†^AFP→US assumes patients received screening for HCC by serum AFP measurements initially and switched to ultrasound if AFP >10 ng/mL; screening method resembles the Alaska Native Health System hepatocellular carcinoma screening program. ^‡^The number of additional tumours that might have been detected at an early stage (i.e. single tumour ≤5 cm in diameter or ≤3 tumours ≤3 cm in diameter) by an US-alone method is unknown; therefore, sensitivity analysis determined the cost/early-stage tumour detected by assuming US-alone method identified 0–100% of the tumours identified by AFP→US at a late stage. ^§^Direct costs of screening discounted at 3%/year (reference year 2012) over a 30-year time horizon. ^¶^Assumes 60% of patients lived in a village without ready US access and required transportation to US facility.

Figure 1 depicts the discounted cost per early-stage tumour detected for the AFP→US and US-alone methods as the percentage of late-stage tumours identified by AFP→US that would potentially have been identified by US-alone at an early stage increases from 0 to 100%. Because we only varied the number of tumours detected by the US-alone method in this analysis, the discounted cost for the AFP→US method remained stable at $36,000/early-stage tumour detected without accounting for travel costs and $59,000 when including travel costs. The discounted cost for US-alone ranged from $38,759 to $81,393/early-stage tumour detected without including travel costs and from $81,064 to $170,235 when including travel costs.

If all persons in our cohort had received HCC screening every 6 months as recommended (i.e. 100% adherence), the total direct discounted costs of the screening program without accounting for travel expenses would have been $556,000 by AFP→US compared with $1.6 million by US-alone (results not shown). Assuming US-alone could have identified all tumours in the cohort at an early stage, full compliance with screening opportunities would have produced a discounted cost without travel expenses of $78,000/early-stage tumour detected.

## Discussion

We compared the cost-effectiveness of HCC screening by using the US-alone method versus AFP→US method in AN people with CHB. Because AASLD guidelines recommend HCC screening by US and discourage using AFP as an HCC screening test, we wanted to compare AFP with the best reported sensitivity of US for detecting HCC. Our results indicate that HCC screening by US-alone might have detected more tumours at an early stage but the overall cost of the screening program would have been more than twice the AFP→US method. Despite using sensitivity estimates for detecting HCC tumours in our model that favoured US as the more effective test compared with AFP, the cost/early-stage tumour detected by the AFP→US method remained lower than the US-alone method, even when we assumed US-alone identified all tumours at an early stage in the sensitivity analysis.

The applicability of our study findings to other health systems must be considered within the unique resource and epidemiologic context of each setting. For example, the cost of a screening program is fixed but the cost/tumour detected is enhanced in regions with a higher incidence of HCC because of a greater potential to detect tumours. This concept is demonstrated by an analysis of an HCC screening program that used both US and AFP at a teaching hospital in Australia (14). The incidence of HCC at the hospital was 2.7%/year and the adjusted cost/tumour detected in 2012 US dollars was approximately $12,600. Because HCC incidence was 10 times higher at that teaching hospital in Australia than in the AN population, the cost/tumour detected was 4 to 8 times lower than either of the methods in our unadjusted model. However, in China, which has a very high prevalence of CHB (7.9% among adults in southeast China), annual per capita spending on health in 2002 was $55 and half of Chinese residents in 1 survey stated that they skipped health services because of cost (15–17). In that context, initial screening by AFP (US$0.60/test in 1997) might be more feasible than lifelong screening by US alone (US$1.20/test in 1997) every 6 months (18).

The effectiveness of a screening program in detecting HCC tumours at an early stage depends on patients’ adherence with recommended screenings. Persons in our cohort received the recommended HCC screening only about half of the time during the 30-year study period. By comparison, 60% of patients at 2 gastroenterology clinics in California with CHB had received optimal screening for HCC over a 3-year period (19). In an HCC screening trial among persons with CHB in China, adherence to screening was 58% over a 5-year period (20). The reasons for suboptimal adherence in our study cohort are unclear. An automated system mails a letter to all AN persons in the CHB registry reminding them to have their blood drawn for AFP measurement, thus eliminating the need for a provider to initiate screening. However, many patients (especially those in rural Alaskan villages) with a normal AFP measurement are notified of the result by mail only. The lack of regular contact with a provider combined with the need for lifelong semi-annual phlebotomy could partly account for the suboptimal adherence.

Our study has several limitations. First, our cost-effectiveness analysis did not take into account the treatments costs for early- and late-stage tumours. We were unable to account for treatment costs because the HCC treatment algorithm is complex, and we lacked data on the probability of receiving the various treatments and their associated outcomes (3). However, it is likely that the US-alone screening approach would have remained more costly even after taking into account treatment costs because of the longer survival of the additional patients that would be detected at an early stage and the eligibility of those patients for more expensive treatments such as liver transplantation. In addition, Medicare reimbursement rates might not reflect the true cost of a screening test and it is likely that the cost of those tests might have declined over time because of increases in availability of the test or operators’ productivity. Although we might have underestimated the overall cost of the screening program, our interpretation of the relative costs of both methods should be unaffected because we used the same test cost estimates for both the AFP→US and US-alone method.

Our study also has strengths. Epidemiologic and clinical data for our cost-effectiveness analysis model were obtained from a large population-based cohort of persons at high risk for HCC who were followed prospectively for up to 30 years. As a result, our model accounts for important real-world factors that affect the cost-effectiveness of a screening program such as patient non-adherence. Moreover, it is unlikely that any persons in our cohort with HCC were missed because the Alaska Native Medical Center is the tertiary referral centre for all persons in our cohort and all cohort patients were cross-referenced with the Alaska Native Tumor Registry. Finally, our sensitivity analysis surrounding several model assumptions enhances the generalisability of our results. For example, our assumption regarding the number of HCC tumours that would have been identified at an early stage by US-alone versus AFP→US relied on papers where the study populations were not representative of our cohort (10,21). However, our sensitivity analysis demonstrated that AFP→US remained more cost-effective than US-alone over a broad range of difference in sensitivity between the 2 HCC screening methods.

Decisions surrounding the optimal method to screen for HCC must balance the cost-effectiveness, as presented here, with other factors not included in this analysis, such as the availability and quality of the screening test (e.g. sensitivity/specificity) (22). The efficacy of AFP as a screening tool for HCC has long been criticised for having a lower sensitivity and specificity than imaging modalities (23,24). But for many of patients in rural Alaska, AFP is the only locally available option for HCC screening, and it could potentially identify patients with CHB at high risk for HCC who could benefit from referral for a liver ultrasound or CT. Thus, public health officials should evaluate the cost-effectiveness of AFP→US to increase access to HCC screening for persons living in remote communities without access to US.

## Appendix

**Appendix Table I:** Demographic and clinical characteristics of the cohort participants with history of hepatitis B virus (HBV) infection and those who developed hepatocellular carcinoma (HCC) – Alaska, 1983–2012.^a^

	Participants with HBV^b^	Participants with HCC
	
Characteristics	N=839	N=21
Ethnic Group		
Eskimo	656 (78%)	21 (100%)
Aleut	87 (10%)	0
Indian	96 (11%)	0
Urban residence	211 (25%)	5 (24%)
Patients alive^c^	553 (66%)	3 (14%)
Mean (median) age of patients alive^c^	56 (55) years	66 (73) years
Mean (median) person-years of follow-up	12 (11) years	10 (9) years
Mean (median) age at HCC diagnosis	–	61 (61) years
HBV Genotype		
A	105 (13%)	2 (10%)
B	44 (5%)	0
C	54 (6%)	9 (43%)
D	387 (46%)	5 (24%)
F	117 (14%)	5 (24%)
H	1 (0.1%)	0
Unknown	131 (16%)	0
Cirrhosis present at HCC diagnosis^d^	Unknown	8 (38%)

a Analysis restricted to males aged >40 years and females aged >50 years.

b Includes patients with HBV infection who subsequently cleared hepatitis B surface antigen.

c As of 12/31/2012.

d Biopsy confirmed.
